# Regulation of Macrophage Polarization in Allergy by Noncoding RNAs

**DOI:** 10.3390/ncrna9060075

**Published:** 2023-12-11

**Authors:** Osamu Ishibashi, Stefan A. Muljo, Zohirul Islam

**Affiliations:** 1 Laboratory of Biological Macromolecules, Graduate School of Agriculture, Osaka Metropolitan University, Sakai 599-8531, Japan; 2 Integrative Immunobiology Section, Laboratory of Immune System Biology, National Institute of Allergy and Infectious Diseases (NIAID), National Institutes of Health (NIH), Bethesda, MD 20892, USA

**Keywords:** macrophage, macrophage polarization, allergy, non-coding RNA

## Abstract

Allergy is a type 2 immune reaction triggered by antigens known as allergens, including food and environmental substances such as peanuts, plant pollen, fungal spores, and the feces and debris of mites and insects. Macrophages are myeloid immune cells with phagocytic abilities that process exogenous and endogenous antigens. Upon activation, they can produce effector molecules such as cytokines as well as anti-inflammatory molecules. The dysregulation of macrophage function can lead to excessive type 1 inflammation as well as type 2 inflammation, which includes allergic reactions. Thus, it is important to better understand how macrophages are regulated in the pathogenesis of allergies. Emerging evidence highlights the role of noncoding RNAs (ncRNAs) in macrophage polarization, which in turn can modify the pathogenesis of various immune-mediated diseases, including allergies. This review summarizes the current knowledge regarding this topic and considers three classes of ncRNAs: microRNAs, long ncRNAs, and circular ncRNAs. Understanding the roles of these ncRNAs in macrophage polarization will provide new insights into the pathogenesis of allergies and identify potential novel therapeutic targets.

## 1. Introduction

Allergies affect millions of people worldwide and are characterized by an excessive type 2 immune response to normally harmless substances, generally known as antigens or allergens, specifically [1,2]. Consequently, this response leads to the development of various allergic symptoms, including asthma, allergic rhinitis, and atopic dermatitis. In the most severe cases, it can result in anaphylaxis and possibly death. According to the World Allergy Organization, the prevalence of allergic diseases has been continuously increasing in the industrialized world [3,4]. In addition, according to the World Health Organization, the number of asthma patients is expected to increase to 400 million by 2025 [4]. The process by which the immune system becomes sensitive to a particular allergen is called sensitization and is typically accompanied by the development of immunoglobulin E (IgE), a specific subclass of antibodies, against the allergen. Sensitization rates to one or more common allergens among schoolchildren are reported to be between 40% and 50% [5]. Since antigen E was isolated from the pollen of common ragweed (*Ambrosia artemisiifolia*) as the first antigen in 1962 [6], a variety of environmental and food allergens have been identified, including 106 allergens that have recently (between January 2019 and March 2021) been accepted by the Allergen Nomenclature Sub-Committee (http://allergen.org/committee.php, accessed on 5 July 2023) [7]. For example, one of the authors (O.I.), together with colleagues, identified *Liposcelis bostrychophila*, a booklouse species commonly found in house dust, as a potent environmental allergen source based on IgE inhibition analysis, which demonstrated that approximately 20% of the studied patients with asthma were sensitized by the *L. bostrychophila*-specific antigen Lip b 1 [8,9]. It should be noted that sensitization to booklice antigens may lead to the misdiagnosis of food-induced allergies. Babaie et al. recently reported that a patient developed anaphylaxis after ingesting oatmeal. However, the results of the skin prick test and serologic testing for oats were negative, and the cause was ultimately identified as booklice contamination of the oatmeal [10]. As insects may become a popular food source in the future, it is important to consider their potential to harbor known and novel allergens [11].

Antihistamines are widely used for symptomatic treatment of many allergic diseases with variable efficacy. Despite the identification of increasing varieties of antigens, there is no fundamental treatment to overcome allergic symptoms except for allergen immunotherapy or desensitization, whereby long-term remission is expected, against a few food and environmental allergens, e.g., cedar pollen [12]. Desensitization therapy is actively investigated because of its clinical potential; however, it harbors the intrinsic risk of inducing severe side effects such as anaphylaxis [13]. Thus, desensitization shots are co-administered with antihistamines. A drug that promotes immune tolerance by targeting macrophages, for instance, could make allergen desensitization safer and even more effective. In 2003, omalizumab, an IgE-blocking antibody received approval from the U.S. Food and Drug Administration (FDA), but it is not approved for all allergic conditions, and it is expensive [14]. Alternatively, blocking antibodies against specific allergens are being developed, but these will be even more expensive for patients that are allergic to multiple allergens. Therefore, an alternative remedy based on a new concept is desirable for this growing patient population.

Immune cells such as mast cells, basophils, dendritic cells, B cells, and specific T-cell subsets are well recognized as key players in allergic reactions. In contrast, to date, macrophages are not commonly associated with allergies. In the future, it would be key for the field to provide credible in vivo evidence that macrophages also play a role in modulating allergy first using mouse models but ultimately in human patients. However, several lines of evidence have recently revealed the crucial role of macrophages in developing and modulating these allergic responses [15,16,17,18,19,20]. For example, macrophages are the most abundant immune cells present in the lungs (approximately 70% of the immune cells) and play a crucial role in asthma caused by environmental-allergen-induced airway inflammation [21,22], suggesting that macrophages, together with other immune cells could play a role in immune responses. Therefore, the role of macrophages in allergic diseases and the mechanism underlying their functional regulation deserve further study.

**Figure 1:**
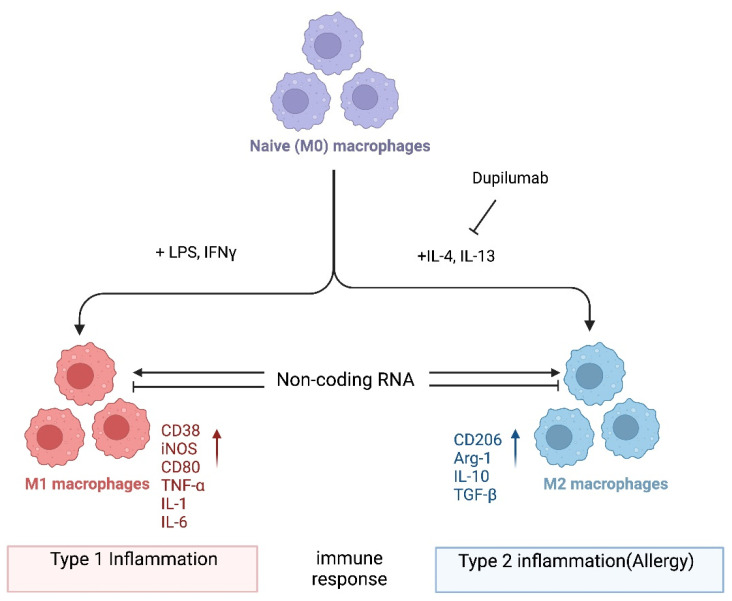
Macrophage polarization. Naïve (M0) macrophages in their inactive state can be polarized into either of two types of activated macrophages with distinct functions, M1 and M2 macrophages (also termed “classically activated” or “alternatively activated” macrophages, respectively), after exposure to certain stimuli. M1 and M2 macrophages are functionally associated with type 1 and type 2 immune reactions, respectively. Several mRNAs and proteins are used as markers to differentiate between these macrophages: i.e., arginase-1 (Arg-1) and CD206 for M2; CD38, CD80, and iNOS for M1 macrophages. However, the criteria for the subclassification of macrophages in vivo in different tissues still require further investigation. Noncoding RNAs might regulate the differentiation of macrophages and/or the function of M1 and M2 macrophages by modifying gene expression programs. Dupilumab is a currently available monoclonal antibody that blocks IL-4 and IL-13 signaling by targeting IL4Rα [35]. This biologic drug is FDA-approved for allergic diseases such as eczema, asthma, and nasal polyps, which result in chronic sinusitis. Hypothetically, its mechanism of action is in part to inhibit M2 polarization. Tralokinumab, another FDA-approved monoclonal antibody, used for the treatment of atopic dermatitis, targets just the cytokine IL-13 (not depicted). Again, it is not well understood which cell types are being affected by this biologic drug. It would be interesting to directly compare dupilumab versus tralokinumab and assess the in vivo effects of each on macrophages and their noncoding transcriptome. Image created with BioRender.com (accessed on 28 November 2023).

In one popular paradigm, macrophages can be divided into two major subclasses, i.e., M1 and M2, based on the inflammatory responses that they mediate, and the process by which macrophages differentiate in response to challenge is called macrophage polarization. Macrophage polarization is determined by the microenvironment (Figure 1). However, the mechanisms underlying in vivo macrophage polarization are complicated and remain largely unclarified, but various intracellular molecules, including signaling molecules and enzymes, and receptors have been shown to regulate macrophage polarization [23,24,25,26,27,28,29,30,31,32,33,34]. For example, it is not known whether dupilumab, a clinically approved biologic drug that blocks IL4Rα signaling [35], leads to an in vivo reduction in M2 macrophages in patients receiving this drug. In the future, it would be important to investigate which immune cells are in fact being inhibited by dupilumab. Alternatively, it is possible that dupilumab switches macrophages into a tolerant state, for instance, by turning on the expression of anti-inflammatory molecules. Thus, it is crucial that the mechanism of action of dupilumab be investigated systematically at the cellular and molecular levels. Here, we provide a consideration of macrophages and noncoding RNAs (ncRNAs). Accumulating evidence has revealed that ncRNAs, a class of functional RNAs not translated into proteins and associated with various pathological events, are associated with both macrophage polarization and allergies. ncRNAs are typically classified into two major types that have distinct functions, i.e., housekeeping ncRNAs and regulatory ncRNAs. The detailed classification of ncRNAs is discussed elsewhere [36,37]. Emerging evidence shows that these ncRNAs play roles in macrophage polarization related to allergies [38,39]. Herein, we summarize the current knowledge on ncRNA-regulated macrophage functions related to allergies, focusing on microRNAs (miRNAs), long ncRNAs (lncRNAs), and circular RNAs (circRNAs), and discuss the possibility of identifying novel potential targets for allergy treatment. In general, we hypothesize that noncoding RNAs could serve as druggable targets to manipulate macrophage plasticity in immune-mediated diseases. With regard to treating allergy specifically, we hypothesize that inhibiting M2 polarization could be a fruitful avenue as exemplified by dupilumab (Figure 1). However, systemic blockade of IL4Rα signaling may have pleiotropic effects in patients, and a target such as a ncRNA that is more specific to M2 could have fewer side effects.

## 2. M1/M2 Macrophage Polarization

Macrophages are evolutionarily ancient white blood cells crucial for the immune system to function properly. They are characterized by high plasticity, which allows them to functionally adapt depending on their microenvironment. Two major macrophage polarization states exist: classically activated macrophages (M1 macrophages) and alternatively activated macrophages (M2 macrophages) (Figure 1). The T helper (Th)1/Th2 balance of T cells affects the balance between the two macrophage polarization states and is critical for maintaining healthy immune functionality [32,33].

M1 macrophages collaborate with Th1 immune responses and play a proinflammatory role in host defense against infection with viruses and intracellular microbes [20,40,41,42]. Polarization of M1 macrophages is typically activated by factors such as interferon (IFN)-γ, a Th1 cytokine, and bacterial products like lipopolysaccharides (LPSs) [24,25,28]. They initiate immune responses by phagocytosing and destroying foreign elements that enter the body, including microorganisms and viruses [40,41,42]. M1 macrophages can also react against endogenous substances in the body. This reaction involves critical physiological functions such as removing cancer cells that are generated in the body [31]; however, they can also cause pathological states such as autoimmune diseases [43,44].

The effector functions of the M1 macrophages are characterized by the production of proinflammatory cytokines such as tumor necrosis factor (TNF)-α, interleukin (IL)-1β, and IL-6, and the expression of inducible nitric oxide synthase (iNOS), which recruits other types of immune cells to the infection or injury site [25,26,27,28,29,30]. In addition to these molecules, some cell-surface proteins, such as CD80 and CD38, serve as M1 macrophage markers [25]. Furthermore, it has been reported that M1 macrophages are involved in forming granulomas, which are masses of immune cells that wall-off infected tissue [45].

In contrast, M2 macrophages are associated with Th2 immune responses and activated by Th2 cytokines, such as IL-4 and IL-13 [2,26,27,28,29,30]. In general, M2 and Th2 cells are associated with allergic reactions. They typically produce anti-inflammatory cytokines, such as IL-10 and transforming growth factor (TGF)-β, which help suppress the immune response and promote wound healing, tissue repair, tissue remodeling, resistance to parasites (e.g., helminths), and tumor growth [46,47]. Additionally, M2 macrophages are involved in clearing apoptotic cells and tissue debris as well as promoting angiogenesis [48]. Compared to M1 macrophages, M2 macrophages show more diverse characteristics. They can be subdivided into several subclasses such as M2a, M2b, M2c, and M2d macrophages based on their functions and the signals they receive [16,28,29] (M2d macrophages are alternatively termed tumor-associated macrophages). However, it is important to know that the validity of the classification and markers remains controversial.

As mentioned above, macrophages in the M1 and M2 states play distinct physiological roles; therefore, an imbalance in these states can lead to various pathological conditions such as chronic inflammation, autoimmune diseases, cancer, metabolic disorders, and infections [34,40,42,44,49,50,51,52,53,54]. Thus, we believe that macrophage polarization is a promising target for drug discovery.

## 3. Association of M2 Macrophages with Immune Tolerance or Suppression in Allergy

The M1 and M2 polarization of macrophages is closely associated with the balance of Th1 and Th2 cells, and it is thought that M2 and Th2 cells are directly linked to allergy [33]. However, macrophages can modulate allergic reactions through their phenotypic plasticity. For example, it has been demonstrated that M2 macrophages regulate allergic responses by suppressing the activity of effector lymphocytes [15,17] and that M2 macrophages can produce resistin-like molecule α (RELMα), which correlates with the appearance of Foxp3-expressing regulatory T cells [32].

As such, various allergic diseases can occur when macrophage functions are dysregulated. Nonetheless, the linkage between macrophage polarization and allergies is still not fully understood. Several studies have explained that dysregulated macrophage functions in the lung and nasal tissues can cause allergic asthma and allergic rhinitis [5,16,18,19,55,56,57,58]. Recent studies have demonstrated the involvement of macrophages in the development of food allergies. For example, chitinase 3-like 1, which is known to be associated with various chronic diseases including allergic disease, plays a pivotal role in M2 macrophage polarization in food allergy [59].

Considering the above-mentioned knowledge, it seems reasonable to propose that targeting macrophage plasticity could be an opportunity to promote immune tolerance and suppress exaggerated immune reactions. However, the anti-inflammatory property of M2 macrophages contradicts previous reports that they are associated with allergic diseases. This contradiction might be explained by differences in the tissue microenvironment and/or their developmental origins, i.e., yolk sac, fetal liver, or adult bone marrow.

## 4. ncRNAs in Macrophage Polarization

As mentioned earlier, ncRNAs are RNA molecules that do not encode proteins and are essential in regulating gene expression at both transcriptional and post-transcriptional levels, which includes epigenetic regulation [60]. There are at least three classes of ncRNAs that regulate gene expression: microRNAs (miRNAs), long ncRNAs (lncRNAs), and circular RNAs (circRNAs). Furthermore, ncRNAs that regulate protein activity have been described [61,62]. While miRNA-mediated regulation of gene expression occurs at the post-transcriptional level, lncRNAs and circRNAs may utilize diverse mechanisms of action. Emerging evidence has highlighted the critical role of ncRNAs in regulating macrophage polarization, which may lead to the development of allergies [63]. Although the mechanisms through which ncRNAs regulate macrophage polarization are diverse and complex, several studies have shown that ncRNAs potentially regulate M1 and M2 macrophage polarization by targeting the regulators of proinflammatory signaling pathways or regulating the expression of anti- or proinflammatory cytokines.

Although published studies have thus far highlighted the functional association between ncRNAs and macrophage polarization or that between ncRNAs and allergic diseases, few reports have described the ncRNA–macrophage polarization–allergy axis. Therefore, in the following sections, we summarize these previous studies on how the individual ncRNA classes are involved in macrophage polarization and how that may relate to allergic diseases.

### 4.1. miRNA-Mediated Regulation of Macrophage Polarization

miRNAs are small (typically ~22 nt in length) ncRNAs that post-transcriptionally regulate gene and protein expression by binding to the 3′-untranslated region of the target mRNAs, which induces mRNA degradation and translational repression [39,64]. Several miRNAs have been demonstrated to regulate macrophage polarization related to allergic diseases (Table 1). For example, it was reported that *miR-155-5p* directly targeted the IL-13 receptor alpha1. Given that IL-13 signaling is associated with M2-mediated allergic diseases like asthma, *miR-155-5p* could regulate the M1/M2 balance [65]. Furthermore, the enhanced proinflammatory response of RAW264.7 macrophage-like cells to IL-33, another proinflammatory cytokine associated with allergic diseases, correlated with increased *miR-155-5p* expression [66]. Thus, it would be of interest to check the *miR-155-5p* expression in macrophages from patients with allergies.

In addition, Jaiswal et al. found that lentiviral overexpression of *let-7c* and *miR-99a-5p* in mouse bone-marrow-derived macrophages (BMDMs) promoted M2 macrophage polarization [67]. Although *let-7c* was previously demonstrated to target C/EBP-δ, thus inhibiting M1 polarization [68], this study identified TNF-α as the target of *miR-99a-5p* as another mechanism to suppress M1 macrophage differentiation [67]. In contrast, it was reported that angiotensin II enhanced M1 macrophage polarization by abrogating *miR-99a-5p* activation [67]. A different study by Wang et al. illustrates that M2 macrophage polarization in allergic rhinitis was promoted by the *miR-202-5p*–Matrilin-2 axis [69]. Furthermore, recently, Lee et al. found, using an ovalbumin-induced allergic asthma mouse model, that the inhibition of *miR-21* suppresses alveolar M2 macrophage polarization [70]. These findings will need to be independently, thoughtfully validated before an allergy drug development strategy is worth considering.

The mannose receptor MRC1/CD206 is expressed in immune cells, and its expression level is pronouncedly elevated in M2 macrophages; therefore, it is generally accepted as an M2 macrophage marker [29]. MRC1/CD206 recognizes an extensive range of surface glycoproteins and plays a crucial role in a variety of immunological events, both physiologically and pathologically [71]. Interestingly, *miR-511-3p* is an miRNA that is transcribed from an intron of the *MRC1* gene. The expressions of *miR-511-3p* and MRC1/CD206 have been shown to be coregulated in macrophages [72,73]. In studies with the MRC1 knockout mouse model in which *miR-511-3p* expression is also deficient, Zhou et al. demonstrated that *miR-511-3p* downregulated M1 macrophage polarization, upregulated M2 macrophage polarization, and protected against cockroach allergen-induced lung inflammation [73]. In addition, it was reported by Do et al. that *miR-511-3p* promoted M2 macrophage polarization and attenuated cockroach-allergen-induced lung inflammation by targeting CCL2 [74]. Alternatively, Heinsbroek et al. demonstrated that *miR-511-3p* regulated intestinal inflammation by controlling macrophage-mediated microbial responses via the indirect upregulation of TLR-4 expression [75]. These findings suggest that *miR-511-3p* regulates macrophage functions and polarization by targeting multiple mRNAs.

Using an allergen-induced asthma knockout mouse model, Chung et al. reported that *miR-451a* negatively affects IL-4-induced M2 macrophage polarization by targeting and silencing the expression of Sirtuin 2 and promoting asthmatic inflammation [76]. Additionally, a few other studies were conducted using ovalbumin-induced allergic asthma mouse models to identify miRNAs in macrophage polarization. For example, Veremeyko et al. demonstrated that *miR-124* expression was upregulated in the lung alveolar macrophages of an ovalbumin-induced allergic lung inflammation mouse model and contributed to the development of M2, but not M1, macrophage polarization [77]. Another study by Shi et al. highlighted the involvement of *miR-142-5p* and *miR-130a-3p* in pulmonary macrophage polarization and asthma airway remodeling in ovalbumin-sensitized mice [78]. Additionally, Su et al. reported that *miR-142-5p* and *miR-130a-3p* functioned by targeting suppressor of cytokine signaling 1 (SOCS1) and peroxisome proliferator-activated receptor γ, respectively [79]. Notably, this study revealed that SOCS1 had a negative impact on the M2 macrophage polarization in mice [79], while the M2 polarization of human macrophages is enhanced by SOCS1 [80]. Again, such contradiction will need to be resolved before ncRNAs can be selected as allergy drug targets.

**Table 1:** MicroRNAs that regulate macrophage polarization in allergy.

miRNA *^1^	Materials Used	Affecting Polarization *^2^	Target	Related Pathophysiology	Reference
*miR-155-5p*	Blood monocytes from healthy donors and the human monocytic cell line THP1	M2 (−)	*IL13R*	Allergic asthma	[65]
*miR-99a-5p*	Mouse bone-marrow-derived macrophages	M1 (−) M2 (+)	*TNF*	Allergic airway inflammation	[67]
*miR-202-5p*	Mucus-derived macrophages from allergic rhinitis patients	M2 (+)	*MATN2*	Allergic rhinitis	[69]
*miR-21-5p*	Ovalbumin-induced allergic asthma mouse model	M2 (+)	Possibly *IRF5*	Allergic asthma	[70]
*miR-511-3p*	Lung macrophages and an allergen-induced lung inflammation mouse model	M1 (−) M2 (+)	*HPGDS*	Allergic lung inflammation	[73]
*miR-511-3p*	Lung macrophages and an allergen-induced lung inflammation mouse model	M1 (−) M2 (+)	*CCL2*	Allergic lung inflammation	[74]
*miR-451a*	Allergen-induced mouse asthma model	M2 (+)	*SIRT2*	Allergic asthma	[76]
*miR-124-3p*	Ovalbumin-induced allergic asthma mouse model	M2 (+)	*CEBPA*	Allergic asthma	[77]
*miR-130a-3p*	Ovalbumin-induced allergic asthma mouse model	M2 (−)	*PPARG*	Allergic asthma	[78,79]
*miR-142-5p*	Ovalbumin-induced allergic asthma mouse model	M2 (+)	*SOCS1*	Allergic asthma	[78,79]

*^1^ miRNAs are indicated as current miRBase identifiers; *^2^ plus and minus signs indicate positive and negative regulation, respectively.

### 4.2. lncRNA-Mediated Regulation of Macrophage Polarization

lncRNAs are long (generally defined to be >200 nt in length) ncRNAs that regulate gene expression at various levels, which include chromatin remodeling, transcriptional regulation, and post-transcriptional regulation [62,63]. Several lncRNAs have been shown to regulate M2 macrophage polarization related to allergies (Table 2). For example, the knockdown of receptor-type tyrosine protein phosphatase ε (PTPRE)-AS1, a lncRNA selectively expressed in IL-4-stimulated macrophages, was shown to promote M2 macrophage activation via the MAPK/ERK 1/2 pathway [81]. Wen et al. recently demonstrated that MIR222HG acts on the *miR146a-5p*/TRAF6/NF-κB axis, leading to the attenuation of macrophage M2 polarization and allergic inflammation in allergic rhinitis [82]. The few studies investigating the lncRNA AK085865 have also highlighted its role in macrophage polarization [83,84]. In particular, the study conducted by Pei et al. showed that AK085865-deficient mice were protected from the allergic airway inflammation induced by Der f 1, a major mite allergen component of *Dermatophagoides farinae* [83]. They also found that AK085865 deletion suppressed M2 macrophage polarization, which subsequently decreased their susceptibility to Der f 1-induced airway inflammation. In addition, Zhang et al. demonstrated that AK085865 specifically interacted with interleukin-enhancer-binding factor (ILF)-2 and functioned as a negative regulator of the ILF2–ILF3-complex-mediated biosynthesis of *miR-192*, which promotes M2 macrophage polarization through the direct targeting of interleukin-1 receptor-associated kinase (IRAK) 1 [84].

lnc-BAZ2B, a lncRNA dominantly expressed in monocytes and significantly upregulated in children with asthma, was also demonstrated to promote M2 macrophage polarization. Mechanistically, lnc-BAZ2B promotes the expression of BAZ2B mRNA by stabilizing its pre-mRNA, leading to enhanced IRF4 expression and M2 macrophage polarization [85]. Another lncRNA reported to regulate the pathological state of allergies is NKILA [86]. This lncRNA was demonstrated to limit the asthmatic airway inflammation, enhancing M2 macrophage polarization and inhibiting the NF-κB pathway in a mouse asthmatic model.

In contrast to many reports on the lncRNA-mediated regulation of M2 macrophage polarization in allergy, there are few reports on the lncRNA–M1 macrophage polarization-allergy axis. One of the few such studies, reported by Jiang et al., describes the contribution of lncRNA MEG8-sponging of *miR-181a-5p* to M1 macrophage polarization via regulating SHP2 expression in a rat model of IgA purpura, which is a type 3 allergic disease triggered by allergens such as drugs, food, or insect bites [87]. In another study, Zhu et al. demonstrated that lncRNA growth-arrest-specific transcript 5 (GAS5) is upregulated in exosomes isolated from the nasal mucus of allergic rhinitis patients and promotes M1 macrophage polarization by restraining autophagy and subsequently activating NF-κB signaling [88].

### 4.3. circRNA-Mediated Regulation of Macrophage Polarization

**Figure 2:**
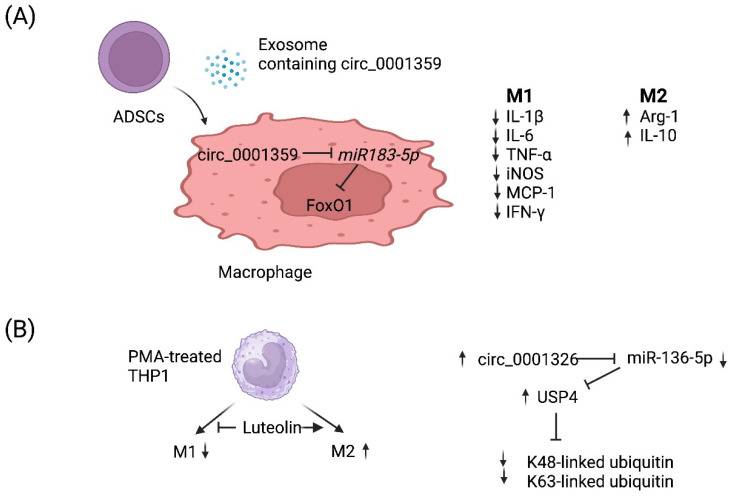
Working models of how two circRNAs regulate M1 vs. M2 macrophage polarization. (**A**) Adipose-derived stem cells (*ADSCs*) secrete exosomes that contain circ_0001359. Upon fusion with macrophages, circ_0001359 is released into the cytoplasm and promotes M2-like macrophage polarization in an ovalbumin-induced asthma mouse model and lipopolysaccharide-induced RAW264.7 macrophages cells as evidenced by Arg-1 and IL-10 expression. In contrast, the expression of the following M1 effector molecules is suppressed by circ_0001359: IL-1β, IL-6, TNF-α, iNOS, MCP-1, and IFN-γ. Mechanistically, circ_0001359 inhibits *miR-183-5p* via base pairing. Since *Foxo1* mRNA is directly repressed by *miR-183-5p*, FoxO1 activity is enhanced as a result and may be in part responsible for reprogramming macrophage cell fate. Image created with BioRender.com (accessed on 28 November 2023). (**B**) Luteolin (a naturally occurring flavonoid found in plants), known for its protective role in asthma, inhibits M1 macrophage polarization and promotes M2 activation in THP-1-derived macrophages. Luteolin-treated THP-1 macrophages induce expression of circ_0001326, inhibiting *miR-136-5p* via base pairing. Consequently, ubiquitin-specific protease 4 (USP4) is upregulated since it is directly repressed by *miR-136-5p*, and ultimately K48-linked and K63-linked ubiquitin is metabolized by USP4 since it is a deubiquitinase enzyme. Image created with BioRender.com (accessed on 28 November 2023).

circRNAs are a recently discovered product of back splicing, and a subset of them do not encode for protein. Thus, they comprise a new category of ncRNAs that form covalently closed circular structures, which make them resistant to degradation by RNA exonucleases [89,90]. Since they are long-lived, a few circRNAs have been shown to act as molecular “sponges” that sequester miRNAs and/or RNA-binding proteins [91]. Although the function of most circRNAs remains poorly understood, a few circRNAs have been demonstrated to regulate the macrophage polarization associated with allergy (Table 2). For example, Shang et al. reported that circ_0001359 was downregulated in ovalbumin-induced asthmatic mice compared with normal mice and circ_0001359-enriched exosomes secreted from adipose-derived stem-cells-attenuated airway remodeling via promoting polarization into M2-like macrophages [92]. Mechanistically, circ_0001359 was shown to regulate macrophage polarization by enhancing FoxO1 signaling via sponging *miR-183-5p* (Figure 2A) [92]. Recently, luteolin, a flavone reported to have a protective role in asthma, was shown to activate M2 and suppress M1 macrophage polarization via upregulating circ_0001326 in the human macrophage cell line THP-1 [93]. The same study also elucidated the underlying mechanism of how circ_0001326 regulates downstream gene expression, including *miR-136-5p* and USP4 (Figure 2B). Finally, it is also conceivable that synthetic circRNAs could be rationally designed to inhibit specific miRNAs to treat diseases such as allergy. Once miRNAs that promote allergy have been identified and validated, then one could simply multimerize the binding sites for miRNA(s) of interest into a synthetic circRNA that will serve to inhibit them and, in turn, allergy.

**Table 2:** Long noncoding and circular RNAs that regulate macrophage polarization in allergy.

LncRNA	Materials Used	Affecting Polarization	Target	Related Pathophysiology	References
PTPRE-AS1	Mouse bone-marrow-derived macrophages	M2 (−)	MAPK/ERK-1/2 pathway	Allergic asthma	[81]
MIR222HG	Mouse RAW264.7 cell line	M2 (−)	*miR146a-5p*/TRAF6/NF-κB axis	Allergic rhinitis	[82]
AK085865	AK085865-deficient mice	M2 (+)	Not determined	Asthmatic airway inflammation	[83,84]
lnc-BAZ2B	Peripheral blood mononuclear cells of asthma patients	M2 (+)	IRF4	Allergic asthma	[85]
NKILA	Asthmatic mouse model	M2 (+)	NF-κB pathway	Asthmatic airway inflammation	[86]
MEG8	Rat peripheral blood cells	M1 (+)	*miR-181a-5p*	IgA purpura (Henoch-Schonlein purpura)	[87]
GAS5	Asthmatic rat model Human ASM culture	M1 (+)	mTORC1/ULK1/ATG13 axis	Allergic rhinitis	[88]
circ_0001359	Asthmatic mouse model	M2 (+)	FoxO1 signaling via sponging *miR-183-5p*	Allergic asthma	[92]
circ_0001326	Human THP-1 cell line	M2 (+) M1 (−)	*miR-136-5p* and USP4	Allergic asthma	[93]

## 5. Therapeutic Implications of the ncRNA–Allergy Axis

Individual ncRNAs regulate the expression of multiple genes and proteins; therefore, they are involved in diverse biological processes including those associated with allergic diseases. Since the dysregulation of macrophage polarization is a primary feature of many allergic diseases, targeting the ncRNAs that regulate macrophage polarization represents a promising therapeutic approach for treating these diseases. As described above, several studies have shown that modulating the expression of specific ncRNAs can alter macrophage polarization and ameliorate allergic symptoms. Of the ncRNAs, miRNAs have particularly attracted attention as promising therapeutic targets of allergic diseases, as is the case for many diseases, e.g., cancer [72].

Of several types of molecules that modulate ncRNA functions, synthetic oligonucleotides such as small interfering (si) RNAs and antisense RNAs are widely used as tools to inactivate specific ncRNAs and are being developed into drugs. For example, patisiran is an FDA-approved siRNA therapeutic [94]. Inotersen is an FDA-approved antisense RNA for the same indication [95]. Thus, in principle, both classes of oligonucleotides can be developed into safe and effective drugs. Synthetic miRNA mimics are frequently used in miRNA studies, as well as antisense RNAs that bind target miRNAs, thus inhibiting their function (antagomirs). For example, a previous in vitro study demonstrated that *miR-155-5p* overexpression via transfection of a synthetic *miR-155-5p* mimic reprogrammed macrophages from the M2 phenotype into M1 phenotype. If this could be accomplished in vivo, then it could be a potential avenue for treating allergies. Conversely, targeting *miR-155-5p* with antisense oligonucleotides restored the defect in M2-like macrophage polarization in rheumatoid arthritis, suggesting that a *miR-155-5p* antagomir may serve as a drug for rheumatoid arthritis treatment [79].

Although several antagomirs and miRNA mimics have been developed as promising oligonucleotide therapeutics, they have yet to reach the market. Conceivably, the major limitation is a difficulty in delivering sufficient amounts of these molecules into the targeted cells. In addition, small RNA molecules that are chemically modified to enhance their specificity and/or stability have been reported to potently induce unfavorable immune responses such as the interferon response in a cell-type-dependent manner [96]. Thus, further research will be necessary to develop oligonucleotide therapeutics with both high efficacy and safety.

In addition to miRNAs, some lncRNAs and circRNAs have been shown to regulate macrophage polarization as mentioned above; thus, they can also be potent therapeutic targets. However, compared with miRNA research, lncRNA and circRNA research has not yet progressed from bench to clinical application. A hurdle to be overcome is the difficulty experienced with the delivery of RNA molecules. As is the case for COVID-19 vaccines, the use of lipid nanoparticles as an RNA carrier is a promising option. In addition, exosomes have been recently highlighted as a promising carrier of RNA molecules including ncRNAs due to their high stability, high biocompatibility, and low immunogenicity [97]. For example, Huang et al. reported that exosomes engineered for the tumor-targeted delivery lncRNA MEG3 had high therapeutic efficacy against osteosarcoma [98]. Exosomes are naturally derived lipid nanoparticles that deliver RNA and/or protein cargo to cells. Therefore, exosomes carrying ncRNAs that regulate macrophage polarization could be less-toxic allergy medications.

## 6. Concluding Remarks

In summary, we discussed how the ncRNA-mediated regulation of macrophage polarization and function could be associated with allergies. However, current knowledge on ncRNA–macrophage–allergy connections is limited; therefore, more mechanistic as well as in vivo studies are needed using either human subjects or animal models that better mimic human allergy. In other human diseases, genome-wide association studies (GWAS) have led to the identification of important drug targets [99,100]. Furthermore, most of the identified associations map to noncoding regions of the human genome [101]. While not all such loci are ncRNA genes, at least a subset of them are [102]. Some are regulatory elements including 3′-untranslated regions (UTRs). It stands to reason that if miRNAs can regulate disease pathogenesis, then variation in their binding site within a 3′ UTR could also have a significant biological effect [103,104]. These targets of natural variation in some cases can also be modulated pharmacologically.

Since M1 and M2 macrophages have been mainly defined and characterized based on the results of simplified in vitro studies, their in vivo roles, where the environment is more heterogeneous and complicated, have yet to be fully elucidated. This might explain why there is no consensus on whether inhibiting or promoting M2 macrophage polarization is associated with allergic diseases. Until this issue is resolved, it will be challenging to rationally design a drug development strategy for allergy. Nonetheless, it is widely accepted that macrophages are an attractive therapeutic target for immune-mediated diseases, and there is no reason to think that allergy is an exception. Moreover, ncRNAs have been considered promising therapeutic targets in addition to biomarkers. Regulatory networks involving cytokines, chemokines, signaling molecules, and transcription factors, as well as epigenetic events such as DNA methylation and histone modifications through methylation and acetylation, are essential for macrophage polarization [54]. Currently, the options available to treat or cure allergic diseases are limited; therefore, many allergic patients continuously receive treatments such as antihistamines, which do not completely relieve their symptoms. In this context, an improved understanding of how these ncRNAs are associated with the complicated networks between macrophage polarization and allergic diseases is required to reveal novel targets for drug discovery and development.

## Data Availability

Not applicable.
